# Supplementary material for: Antimicrobial Resistance of Salmonella and Characterization of Two Mcr-1-Harboring Isolates from Pork Products in Guangdong, China
Source: Foods. 2025 Aug 22;14(17):2933. doi: 10.3390/foods14172933 (PMC12427947; doi:10.3390/foods14172933)
Supplement: Supplementary file 1 [file foods-14-02933-s001.zip › foods-3824030 Supplementary File S1.pdf]

Table S1. Monthly *Salmonella* Positivity Rate in Two Pig Slaughterhouses in Guangdong Province, 2023

Collection time	Number of samples in slaughterhouse one	Number of samples in slaughterhouse two	Total number of samples in these two slaughterhouses	Number of samples positive for *Salmonella*	Percentage of positive samples (%)
January	26	31	57	4	7.0
March	26	31	57	9	15.8
May	26	31	57	8	14.0
June	26	31	57	18	31.6
July	26	31	57	16	28.1
August	26	32	58	21	36.2
October	25	32	57	10	17.5
December	25	32	57	6	10.5
Total	206	251	457	92	20.1

Table S2. The antimicrobial resistance spectrums of 92 *Salmonella* isolates

Antimicrobial resistance spectrum	Co-resistant drug classes	Number (rate/ %)
TET-SUL	2	18 (19.6)
TET-SUL-AMP-NAL-CIP-OFX	4	12 (13.0)
TET	1	10 (10.9)
TET-SUL-AMP-STR-CHL-KAN	5	8 (8.7)
TET-SUL-AMP-STR-CHL	5	7 (7.6)
TET-SUL-AMP-NAL-CIP-OFX-GEN	5	5 (5.4)
TET-SUL-AMP	3	4 (4.3)
TET-SUL-AMP-STR-CHL-NAL	6	3 (3.3)
TET-SUL-AMP-NAL-CIP-OFX-KAN	5	2 (2.2)
TET-SUL-AMP-STR-CHL-NAL-CIP-OFX-GEN	6	2 (2.2)
TET-SUL-AMP-AZI	4	1 (1.1)
TET-SUL-AMP-GEN	4	1 (1.1)
TET-SUL-AMP-STR-CHL-AZI	6	1 (1.1)
TET-SUL-AMP-STR-CHL-NAL-KAN	6	1 (1.1)
TET-SUL-AMP-STR-CHL-GEN-FOS	6	1 (1.1)
TET-SUL-AMP-STR-CHL-NAL-CIP-OFX	6	1 (1.1)
TET-SUL-AMP-STR-CHL-NAL-GEN-KAN	6	1 (1.1)
TET-SUL-AMP-STR-CHL-NAL-CIP-OFX-GEN-AZI	7	1 (1.1)
TET-SUL-AMP-STR-CHL-NAL-CIP-OFX-GEN-AZI-FOS	8	1 (1.1)
TET-SUL-AMP-STR-CHL-NAL-CIP-OFX-GEN-KAN-CTX-FEP-COL	7	1 (1.1)
TET-SUL-AMP-STR-CHL-NAL-CIP-OFX-GEN-KAN-CTX-FEP-AMK-TGC-COL	8	1 (1.1)

Note: CIP: ciprofloxacin, OFX: ofloxacin, NAL: nalidixic acid, FEP: cefepime, CTX: cefotaxime, AMP: ampicillin, TGC: tigecycline, TET: tetracycline, KAN: kanamycin, AMK: amikacin, GEN: gentamicin, STR: streptomycin, SUL: sulfisoxazole, FOS: fosfomycin, CHL: chloramphenicol, AZI: azithromycin, and COL: colistin.

Table S3. Antimicrobial Resistance Among *Salmonella* and *Salmonella* serotypes Isolated from Different Pig Slaughterhouses in Guangdong, China

Antimicrobials	Number of antimicrobial-resistant isolates among (%)											
	Slaughterhouse one						Slaughterhouse two					
	All *Salmonella* in slaughterhouse one (n=54)	Typhimurium (n=18)	Rissen (n=13)	London (n=13)	Derby (n=8)	Kentucky (n=2)	All *Salmonella* in slaughterhouse two (n=34)	Rissen (n=16)	Typhimurium (n=11)	London (n=7)	Derby (n=3)	Corvallis (n=1)
Folate pathway inhibitors												
Sulfisoxazole	46 (85.2)	18 (100.0）	9 (69.2)	9 (69.2)	8 (100.0)	2 (100.0)	25 (65.8)	8 (50.0)	9 (81.8)	5 (71.4)	2 (66.7)	1 (100.0)
Quinolones												
Nalidixic acid	23 (42.6)	10 (55.6)	2 (15.4)	2 (15.4)	7 (87.5)	2 (100.0)	8 (21.1)	1 (6.3)	6 (54.5)	1 (14.3)	0 (0)	0 (0)
Ofloxacin	19 (35.2)	6 (33.3)	2 (15.4)	2 (15.4)	7 (87.5)	2 (100.0)	7 (18.4)	0 (0)	6 (54.5)	1 (14.3)	0 (0)	0 (0)
Ciprofloxacin	19 (35.2)	6 (33.3)	2 (15.4)	2 (15.4)	7 (87.5)	2 (100.0)	7 (18.4)	0 (0)	6 (54.5)	1 (14.3)	0 (0)	0 (0)
β-Lactam												
Ampicillin	38 (70.4)	18 (100.0）	8 (61.5)	2 (15.4)	8 (100.0)	2 (100.0)	16 (42.1)	3 (18.8)	8 (72.7)	2 (28.6)	2 (66.7)	1 (100.0)
Cefotaxime	2 (3.7)	0 (0)	0 (0)	0 (0)	0 (0)	2 (100.0)	0 (0)	0 (0)	0 (0)	0 (0)	0 (0)	0 (0)
Cefepime	2 (3.7)	0 (0)	0 (0)	0 (0)	0 (0)	2 (100.0)	0 (0)	0 (0)	0 (0)	0 (0)	0 (0)	0 (0)
Tetracyclines												
Tetracycline	50 (92.6)	18 (100.0）	13 (100.0)	9 (69.2)	8 (100.0)	2 (100.0)	32 (84.2)	13 (81.3)	11 (100.0）	5 (71.4)	2 (66.7)	1 (100.0)

Tigecycline	1 (1.9)	0 (0)	0 (0)	0 (0)	0 (0)	1 (50.0)	0 (0)	0 (0)	0 (0)	0 (0)	0 (0)	0 (0)
Phenicols												
Chloramphenicol	25 (46.3)	13 (72.2)	6 (46.2)	2 (15.4)	2 (25.0)	2 (100.0)	4 (10.5)	1 (6.3)	2 (18.2)	0 (0)	0 (0)	1 (100.0)
Aminoglycosides												
Streptomycin	25 (46.3)	13 (72.2)	6 (46.2)	2 (15.4)	2 (25.0)	2 (100.0)	4 (10.5)	1 (6.3)	2 (18.2)	0 (0)	0 (0)	1 (100.0)
Gentamicin	14 (25.9)	1 (5.6)	1 (7.7)	2 (15.4)	8 (100.0)	2 (100.0)	0 (0)	0 (0)	0 (0)	0 (0)	0 (0)	0 (0)
Amikacin	1 (1.9)	0 (0)	0 (0)	0 (0)	0 (0)	1 (50.0)	0 (0)	0 (0)	0 (0)	0 (0)	0 (0)	0 (0)
Kanamycin	12 (22.2)	7 (38.9)	3 (23.1)	0 (0)	0 (0)	2 (100.0)	2 (5.3)	0 (0)	2 (18.2)	0 (0)	0 (0)	0 (0)
Polymyxins												
Colistin	2 (3.7)	0 (0)	0 (0)	0 (0)	0 (0)	2 (100.0)	0 (0)	0 (0)	0 (0)	0 (0)	0 (0)	0 (0)
Macrolides												
Azithromycin	3 (5.6)	0 (0)	1 (7.7)	2 (15.4)	0 (0)	0 (0)	1 (2.6)	1 (6.3)	0 (0)	0 (0)	0 (0)	0 (0)
Fosfomycins												
Fosfomycin	2 (3.7)	0 (0)	1 (7.7)	1 (7.7)	0 (0)	0 (0)	0 (0)	0 (0)	0 (0)	0 (0)	0 (0)	0 (0)
Carbapenems												
Meropenem	0 (0)	0 (0)	0 (0)	0 (0)	0 (0)	0 (0)	0 (0)	0 (0)	0 (0)	0 (0)	0 (0)	0 (0)
Multidrug resistance	38 (70.4)	18 (100.0）	8 (61.5)	2 (15.4)	8 (100.0)	2 (100.0)	16 (42.1)	3 (18.8)	8 (72.7)	2 (28.6)	2 (66.7)	1 (100.0)

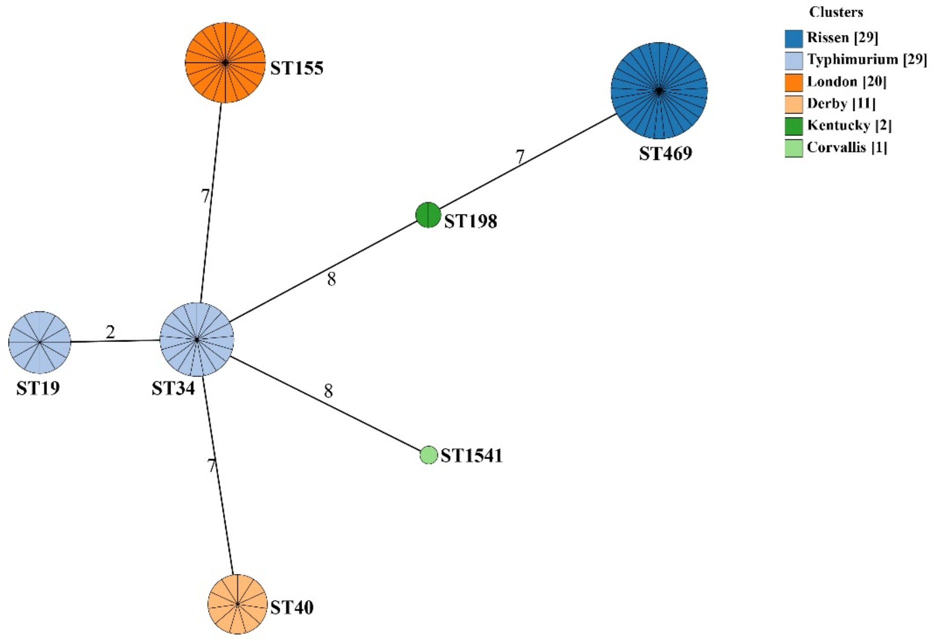

Figure S1. Minimum spanning tree of *Salmonella* isolates by multi-locus sequence typing. Each node in the tree corresponds to a unique sequence type (ST), with the size of the node reflecting the number of isolates associated with that ST. The branch length connecting nodes represents the genetic distance calculated from nucleotide variances in seven conserved housekeeping genes of *Salmonella*.

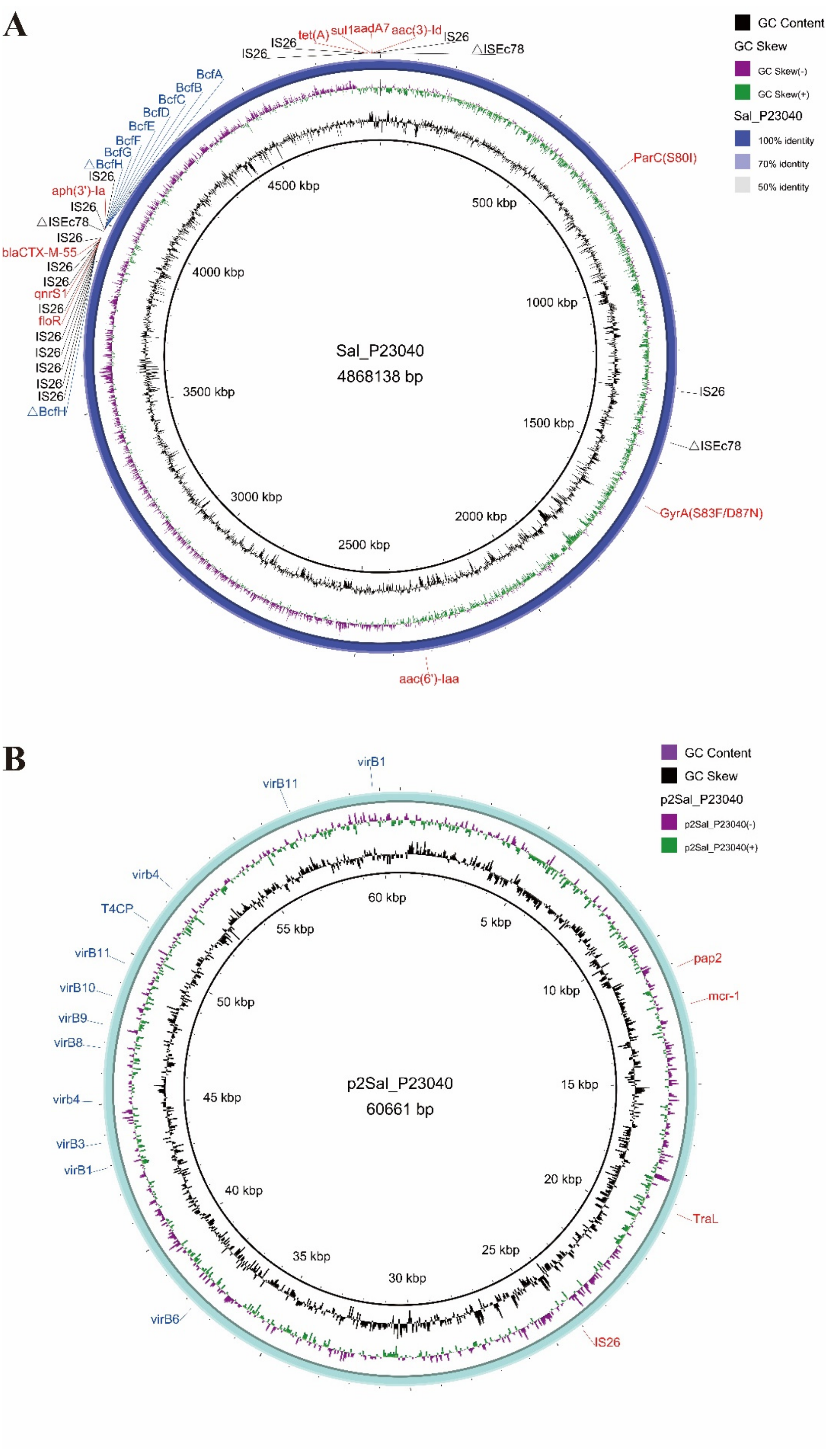

Figure S2. Schematic diagram of the whole genome sequence of isolate Sal_P23040. **A.** Whole genome sequence diagram of isolate Sal_P23040. chromosome. **B.** Whole genome sequence diagram of plasmid p2Sal_P23040.
